# Effects of Enzyme Volumes on Hydrolysis and Fermentation for Ethanol Production From Leftover Cooked Rice

**DOI:** 10.3389/fbioe.2021.631089

**Published:** 2021-05-24

**Authors:** Xikai Chen, Yujia Zhao, Boyang Chen, Wang Su, Zhengxian Zhang, Yanxu Liu, Xiaobin Xu, Junhong Tang, Pingzhi Hou, Wei Han

**Affiliations:** ^1^College of Materials and Environmental Engineering, Hangzhou Dianzi University, Hangzhou, China; ^2^Zhejiang Jinhuanbao Biotechnology Company Limited, Huzhou, China; ^3^School of Material Science and Engineering, Shanghai Jiao Tong University, Shanghai, China; ^4^College of Electronics and Information, Hangzhou Dianzi University, Hangzhou, China; ^5^College of Environmental and Chemical Engineering, Shanghai University, Shanghai, China; ^6^School of Automation, The Belt and Road Information Research Institute, Hangzhou Dianzi University, Hangzhou, China; ^7^The Sci-Tech Academic Institute, Hangzhou Dianzi University, Hangzhou, China

**Keywords:** ethanol fermentation, glucoamylase, leftover cooked rice, reducing sugar, hydrolysis

## Abstract

This study aimed to utilize the enzymatic hydrolysis of leftover cooked rice (LCR) for fermentative ethanol production. Effect of glucoamylase volumes (V1: 5 U/g, V2: 25 U/g, and V3: 50 U/g) on the performance of LCR hydrolysis was also evaluated. It was found that the highest chemical oxygen demand (COD) of 77.5 g/L and reducing sugar (RS) of 34.6 g/L were achieved at V3. The LCR hydrolyzate obtained from enzymatic hydrolysis was then used as feedstock for ethanol fermentation. Higher ethanol production was obtained when RS increased from 18.7 g/L (V1) to 23.2 g/L (V2). However, lower ethanol production was found when RS further increased to 34.6 g/L (V3) probably because too high RS concentration led to the inhibition on the yeast. The maximum ethanol production and yield were 21.1 g/L and 0.3 g ethanol/g LCR, respectively. The LCR could be a promising substrate for fermentative ethanol production for industrial application.

## Graphical Abstract

A combination bioprocess for ethanol production via hydrolysis and fermentation.

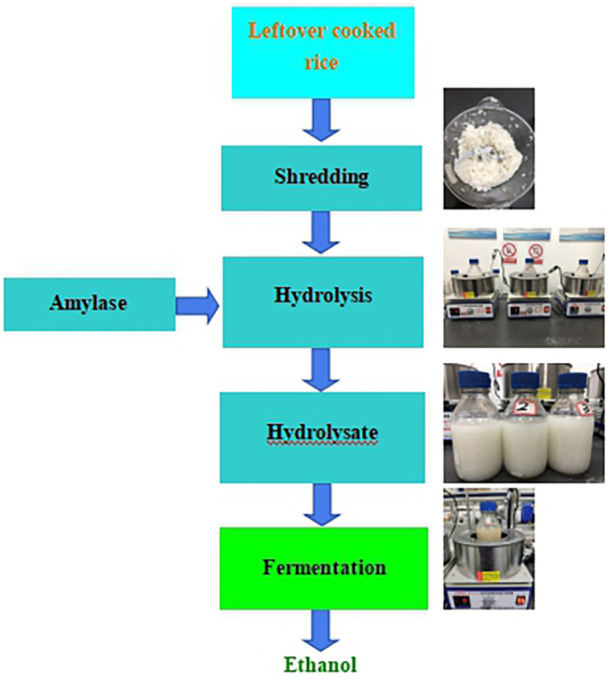

## Introduction

Due to the depletion of the fossil fuel and emission of the greenhouse gases, it is urgent to look for the renewable and clean alternative fuels to satisfy the global energy demands (Florentino-Madiedo et al., 2018; Li et al., 2019). Ethanol is considered to be one of the promising substitutes because it could meet the above requirements (Balat and Balat, 2009; Wychen and Laurens, 2013). Biological ethanol production from various types of substrates (corn, wheat, etc.) has been realized (Kim and Dale, 2004). However, these substrates are not the prospective choices for ethanol production since the global demand for food has not been satisfied (Condon et al., 2015). So, utilization of organic wastes for fermentative ethanol production has gained a great of attentions (Himmel et al., 2007).

The cooked rice is one of the most important staple foods, especially in China and East Asia countries (Liu et al., 2013; Han et al., 2017). It was reported that around 34 million tons of cooked rice were wasted per year in China (Zhao et al., 2019). The leftover cooked rice (LCR), which is rich in carbohydrate (starch), should be a good feedstock for bio-ethanol generation (Chen et al., 2016). However, it is difficult for microorganisms to directly utilize starch for fermentative ethanol production (Xu and Wang, 2017). Pre-treatment is required to make the LCR to be the fermentable nutrients (Rodriguez et al., 2015; Uçkun Kiran and Liu, 2015). There are three main methods used for biomass or organic solid waste pretreatments (Karimi and Taherzadeh, 2016; Lyu et al., 2019), including physical (shredding and heating), chemical (acid and alkaline), and biological (enzyme) methods. Enzymatic hydrolysis is regarded as a better choice compared to the others because it is performed at the mild condition and has no corrosion problem (Aditiya et al., 2016). However, information about enzymatic hydrolysis of LCR and ethanol fermentation from the LCR hydrolyzate is limited.

Therefore, this study aimed to utilize the enzymatic hydrolysis of LCR as substrate for fermentative ethanol production. Effect of glucoamylase volumes (V1: 5 U/g, V2: 25 U/g, and V3: 50 U/g) on the performance of LCR hydrolysis was investigated. RS produced in the LCR hydrolyzate was then fermented by the yeast for ethanol production. The efficiency of ethanol fermentation was also examined. It was expected that this work could provide a practical solution for LCR treatment and ethanol production in China and East Asia countries.

## Materials and Methods

**FIGURE 1:**
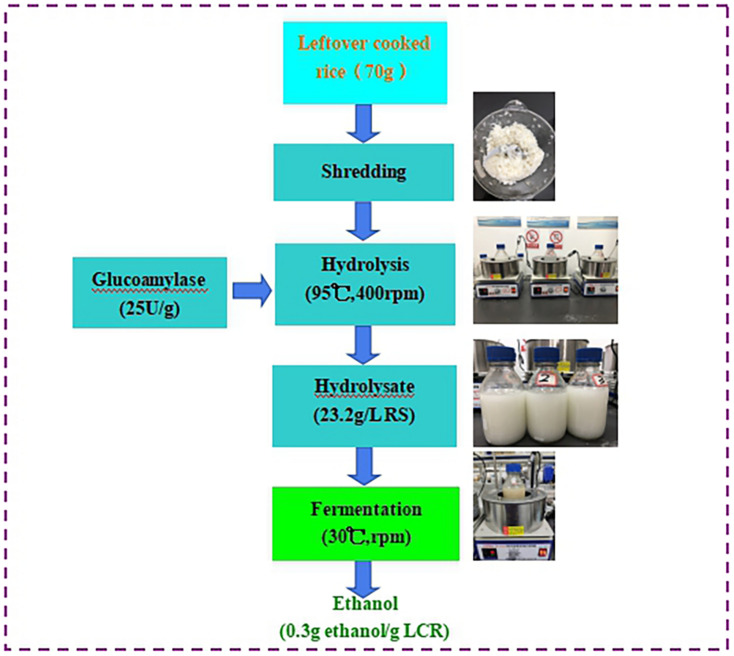
Schematic diagram and materials balance of fermentative ethanol production from enzymatic hydrolysis of leftover cooked rice with the enzyme volume of 25 U/g (V2). No sterilization and additional pH buffer and supplement were applied in this study.

The schematic diagram of fermentative ethanol production from enzymatic hydrolysis of LCR was shown in Figure 1. The separated hydrolysis and fermentation process was selected in this study because of different temperatures required for enzymatic hydrolysis (95°C) and ethanol fermentation (30°C). It should be emphasized that sterilization and additional pH buffer and supplement were not applied. This is crucial for industrial application because the ethanol production cost could be inevitably reduced.

### Raw Materials

The LCR was collected from the canteen of Hangzhou Dianzi University in Hangzhou, China. Its nutrients were analyzed and showed in Table 1. The LCR was first ground into the smaller size by the blender and then used for enzymatic hydrolysis (Farah Amani et al., 2017). Different from complex substrates (such as food waste), a combination enzymes (protease and cellulase) are not necessary in this study to break the LCR into fermentable sugars. The glucoamylase used for enzymatic hydrolysis was provided by the Ningxia Chemicals Ltd. (Ningxia, China). The optimal temperature range of the enzyme was 90–100°C with activity of 7 × 104 U/mL. The dry yeast *Saccharomyces cerevisiae* for ethanol fermentation was supplied by Zhejiang Jinhuanbao Biotechnology Company Limited (Huzhou, China). It was pre-cultivation with glucose and tap water (100:1:1,000, w/w/w) at the temperature of 30°C for 30 min. Then, the produced soluble yeast was utilized as inoculum for ethanol fermentation.

**TABLE 1:** The nutrient of the leftover rice used in this study.

Nutrient	Content (100 g/LR)	Unit
Energy	121	kcal
Carbohydrate	23.7	g
Moisture	60	g
Fat	0.29	g
Protein	3.1	g
Cellulose	0.28	g
K	23	mg
Na	139	mg

### LCR Hydrolysis

Leftover cooked rice hydrolysis was carried out in the bioreactor with effective volume of 500 mL. The LCR with 70 g was taken into the bioreactor with addition of tap water to the level of 500 mL. Effect of glucoamylase volumes (V1: 5 U/g, V2: 25 U/g, and V3: 50 U/g) on the performance of LCR hydrolysis was evaluated. The hydrolysis conditions were 95°C and 400 rpm, respectively. When the reducing sugar (RS) reached a maximum and became stable, the mixture was filtrated by the filtration paper to get the LCR hydrolyzate.

### Fermentative Ethanol Production From LCR Hydrolyzate

Utilization of LCR hydrolyzate as substrate for ethanol fermentation was conducted in another bioreactor. Inoculum (10 mL pre-cultivated yeast solution) was injected into the bioreactor with the produced LCR hydrolyzate. The ethanol fermentation was operated at the conditions of 30°C and 400 rpm agitation speed. Around 10 mL samples were taken at the intervals for ethanol, chemical oxygen demand (COD) and RS analysis.

### Analytical Methods

The RS was determined by the DNS method, while the COD concentration was measured based on the Standard Methods (Franson, 1995). The produced ethanol was analyzed by the gas chromatography which was equipped with the flame ionization detector (FID). Nitrogen was used as the carrier gas. The detailed information for the ethanol analysis was provided in our published study (Han et al., 2019).

## Results

### Enzymatic Hydrolysis of LCR

**FIGURE 2:**
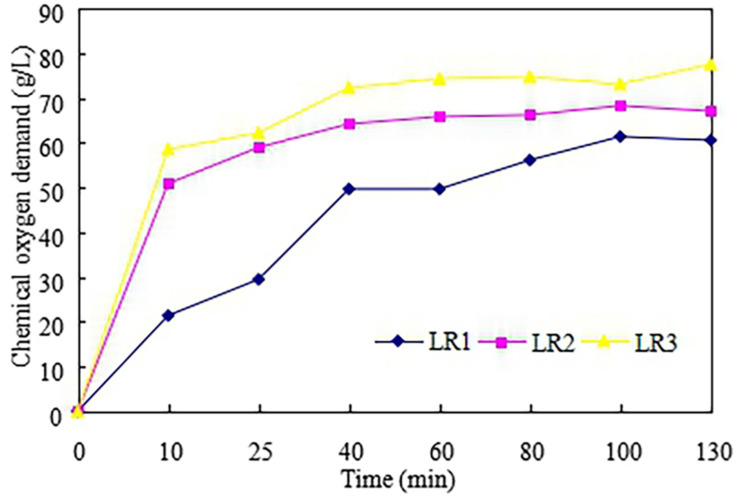
Production of the chemical oxygen demand in the enzymatic hydrolysis of leftover cooked rice.

In this part, LCR was hydrolyzed by glucoamylase to produce the LCR hydrolyzate which was then used as substrate for ethanol fermentation. Effect of enzyme volumes on the hydrolysis efficiency was also evaluated. Figure 2 described the production of COD in the enzymatic hydrolysis of LCR. It can be observed that COD enhanced from V1 to V3 and the highest COD concentration of 77.5 g/L was obtained with V3. This is because higher enzyme volume could be benefit for enzymatic hydrolysis. It is similar to Ali Mandegari study (Ali Mandegari et al., 2017) that the hydrolysis rate was determined by the added amount of the enzyme. However, the enzyme volume of V3 (50 U/g) was twice higher than V2 (25 U/g) and 10 times than V1 (5 U/g), while the COD production increased 10.6% for V2 and 28% for V3 compared to V1. Ye et al. (2018) found that the enzyme activity might be inhibited by the glucose produced during hydrolysis. So, the enzyme volume of 5 U/g (V1) could to be the economic choice for LCR hydrolysis.

**FIGURE 3:**
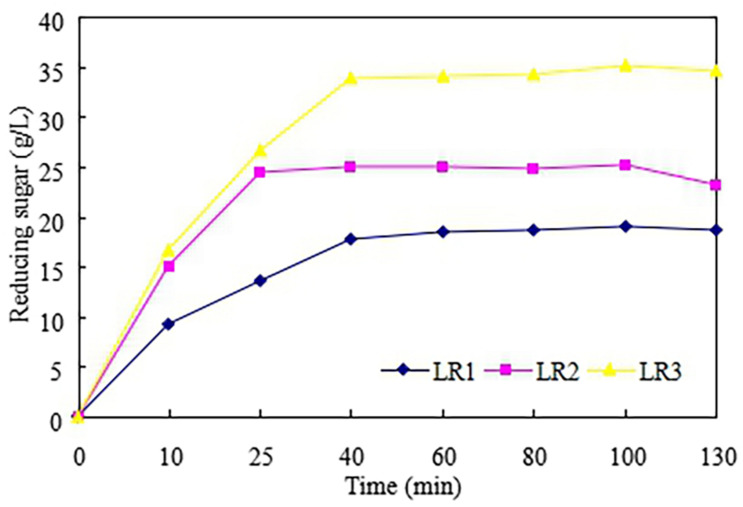
Production of reducing sugar in the enzymatic hydrolysis of leftover cooked rice.

Figure 3 showed the production of the RS in the enzymatic hydrolysis of LCR with different enzyme volumes (V1–V3). Similar to the COD production, the RS increased with increasing of enzyme volumes. The highest RS obtained for V1–V3 were 18.7, 23.2, and 34.6 g/L, respectively. This result was similar to Farah Amani (Farah Amani et al., 2017) who reported that the rate of hydrolysis enhanced with increasing of enzyme volumes used. The kinetic models for enzyme volumes and COD/RS were shown in Table 2. It was found that enzyme volumes (x) and COD/RS (y1/y2) have a good linear relationship which could be expressed as y1 = 188.69x + 58.3 (*R*^2^ = 0.994) and y2 = 178.77x + 15.9 (*R*^2^ = 0.967), respectively.

**TABLE 2:** Relationship between enzyme volumes and COD/RS in the LCR hydrolyzate.

	V1	V2	V3	Kinetic model (y1/y2: COD/RS, x: enzyme volumes)	*R*^2^
COD	60.6	67	77.5	Y1 = 188.69x + 58.3	0.994
RS	18.7	23.2	34.6	Y2 = 178.77x + 15.9	0.967

### Ethanol Fermentation From LCR Hydrolyzate

**FIGURE 4:**
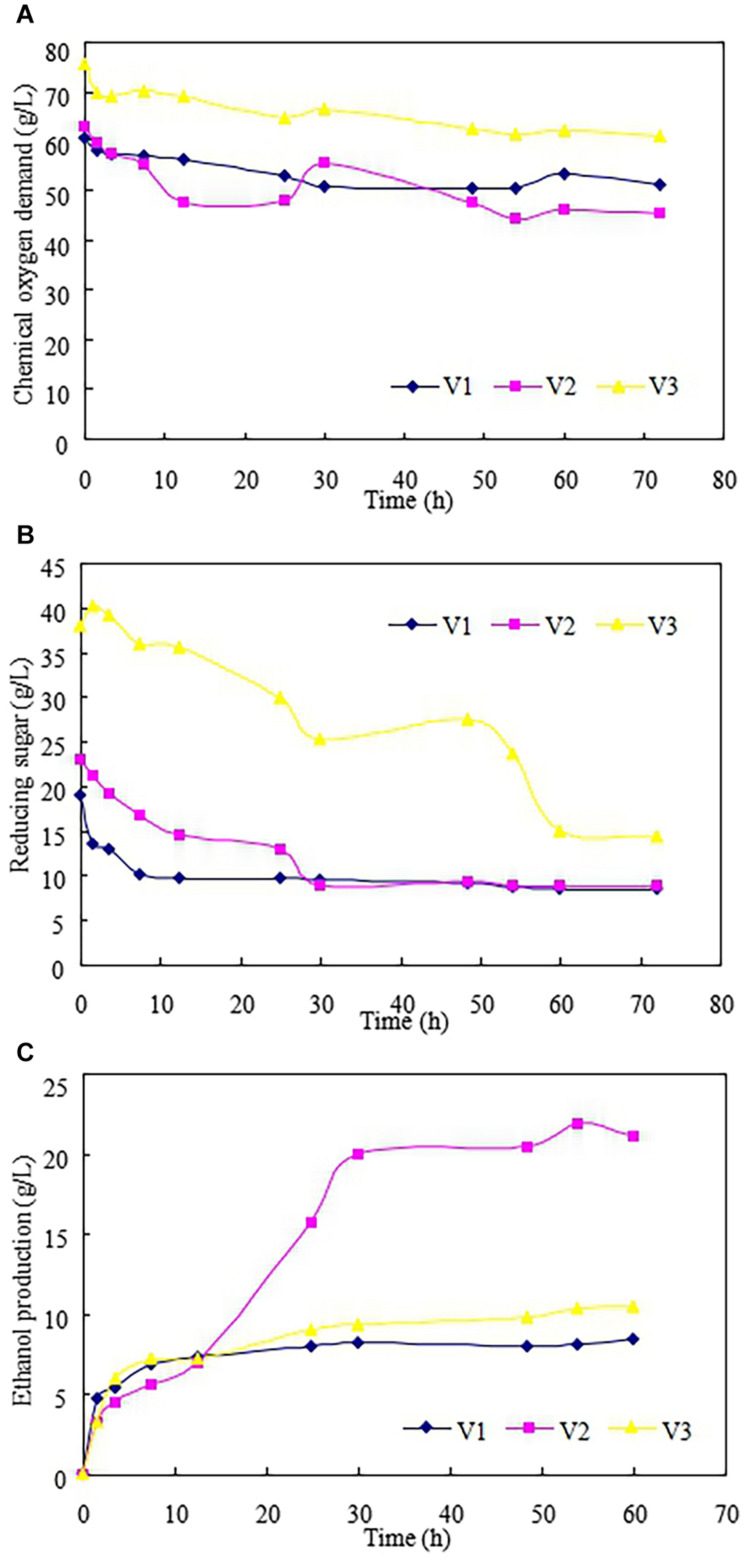
Fermentative ethanol production from enzymatic hydrolysis of leftover cooked rice.

Figure 4 showed the performance of ethanol fermentation from LCR hydrolyzate. No additional pH buffer and supplement was applied in this study. Also, the sterilization was not needed because the further practical application could be taken into account (Leung et al., 2012). As shown in Figure 4A, the COD decreased at the beginning of the ethanol fermentation since the RS contained in the LCR hydrolyzate was consumed by the yeast. Then, the COD stabilized at around 50 h as the ethanol was produced (Ali Mandegari et al., 2017). The final COD concentrations for V1-V3 were 51, 45.2, and 60.7 mg/L, respectively.

Figure 4B described the RS consumed in the fermentative ethanol production. No lag period was observed for all the batches since the yeast used in this study had good adaptation (Jelena et al., 2012). The RS was consumed and the ethanol produced (Figure 4C) at the beginning of the fermentation. This result is different from Kim’s study (Ye et al., 2018) that the ethanol production from enzymatic hydrolysis of food waste was not found until 20 h after inoculation of yeast. This is probably because the filtration after enzymatic hydrolysis was not applied in Kim’s study (Ye et al., 2018) and the inhibitors (such as oil) inhibited the activity of the yeast for ethanol production. In this study, the ethanol production increased with increasing of RS concentration (V1 and V2) and the highest ethanol production of 21.1 g/L was achieved with V2. However, it was found that the ethanol production became lower when the RS concentration further increased. This is probably because the RS concentration is too high to inhibit the activity of yeast for ethanol production (Manfredi et al., 2018). In the batch V3, the RS further decreased from 30 h, while the COD and ethanol were no longer increased. It was indicated that the RS was consumed by the yeast for anabolic reaction (microorganism growth), not for catabolic metabolism (ethanol production) (Erdei et al., 2013). The kinetic performance of ethanol fermentation from LCR hydrolyzate was described in Table 3. It was observed that the maximum productivity of ethanol and ethanol yield of 0.84 g/L/h and 0.3 g ethanol/g LCR could be calculated, respectively.

**TABLE 3:** Kinetic values by yeast for fermentative ethanol production from LCR hydrolyzate with the enzyme volume of 25 U/g (V2).

Kinetic parameter	Value	Unit
Doubling time (t_h_)	30	h
Reducing sugar consumed	14.15	g/L
Maximum productivity of ethanol	0.84	g/L/h
Ethanol yield (Y_e/LCR_)	0.3	g ethanol/g LCR

## Discussion

Some studies reported the comparison of bioethanol production between separate hydrolysis and fermentation (SHF) and simultaneous saccharification and fermentation (SSF). In this study, SHF should be the better choice for bioethanol production from LCR due to the following two reasons. First, the temperatures for hydrolysis (95°C) and fermentation (30°C) are different. Higher hydrolysis efficiency could be obtained with SHF. Second, the inhibitors (such as oil) for ethanol fermentation could be removed by filtration after hydrolysis with SHF. Similar results have been found by other researchers. Ye et al. (2018) got the highest ethanol yields of 0.31 and 0.43 g ethanol/g TS from food waste for SSF and SHF, respectively. F Alfani et al. (2000) compared the bioethanol production from wheat straw with SSF and SHF. It was found that the ethanol yield was 68% of the theoretical yield for SSF, while the theoretical yield for SHF was 81%.

The ethanol yield obtained from this study was 0.3 g ethanol/g LCR which was equivalent to 0.75 g ethanol/g dry LCR (Figure 1). To our knowledge, this is the first study to report the fermentative ethanol production from LCR. This study also demonstrated that the LCR is a promising substrate for ethanol fermentation.

## Conclusion

Utilization of LCR hydrolyzed by different glucoamylase volumes for fermentative ethanol production was investigated in this study. The highest COD of 77.5 g/L and RS of 34.6 g/L in the LCR hydrolyzate were obtained with the enzyme volume of 50 U/g. In the ethanol fermentation, no lag period was observed and the doubling time was 30 h. The ethanol productivity and ethanol yield were 0.84 g/L/h and 0.3 g ethanol/g LCR, respectively. To our knowledge, this is the first study to report the fermentative ethanol production from LCR and provide the basic information for further industrial application.

## Data Availability Statement

The original contributions presented in the study are included in the article/supplementary material, further inquiries can be directed to the corresponding author/s.

## Author Contributions

WH, XC, and YZ designed the experiments. YL and BC performed the biological experiments. XX, JT, and PH analyzed the data. WS and ZZ assisted with the ethanol content measurement. WH and JT supervised the manuscript. XC, YZ, and WH wrote the manuscript with contributions from all authors. All authors contributed to the article and approved the submitted version.

## Conflict of Interest

XC, YZ, WS, ZZ, and WH were employed by the company Zhejiang Jinhuanbao Biotechnology Company Limited. The remaining authors declare that the research was conducted in the absence of any commercial or financial relationships that could be construed as a potential conflict of interest.
